# Effects of Long-Term DHA Supplementation and Physical Exercise on Non-Alcoholic Fatty Liver Development in Obese Aged Female Mice

**DOI:** 10.3390/nu13020501

**Published:** 2021-02-03

**Authors:** Jinchunzi Yang, Neira Sáinz, Elisa Félix-Soriano, Eva Gil-Iturbe, Rosa Castilla-Madrigal, Marta Fernández-Galilea, J. Alfredo Martínez, María J. Moreno-Aliaga

**Affiliations:** 1 Center for Nutrition Research and Department of Nutrition, Food Sciences and Physiology, University of Navarra, 31008 Pamplona, Spain; jyang@alumni.unav.es (J.Y.); nsainz@unav.es (N.S.); efelix@alumni.unav.es (E.F.-S.); egil.5@alumni.unav.es (E.G.-I.); rcastilla@alumni.unav.es (R.C.-M.); mfgalilea@unav.es (M.F.-G.); jalfmtz@unav.es (J.A.M.); 2 Navarra’s Health Research Institute (IdiSNA), 31008 Pamplona, Spain; 3 CIBERobn Physiopathology of Obesity and Nutrition, Carlos III Health Institute, 28029 Madrid, Spain

**Keywords:** obesity, aging, non-alcoholic fatty liver, omega-3 fatty acids, exercise, lipogenesis, fatty acid oxidation, inflammation, ER stress, autophagy

## Abstract

Obesity and aging are associated to non-alcoholic fatty liver disease (NAFLD) development. Here, we investigate whether long-term feeding with a docosahexaenoic acid (DHA)-enriched diet and aerobic exercise, alone or in combination, are effective in ameliorating NAFLD in aged obese mice. Two-month-old female C57BL/6J mice received control or high fat diet (HFD) for 4 months. Then, the diet-induced obese (DIO) mice were distributed into four groups: DIO, DIO + DHA (15% dietary lipids replaced by a DHA-rich concentrate), DIO + EX (treadmill running), and DIO + DHA + EX up to 18 months. The DHA-rich diet reduced liver steatosis in DIO mice, decreasing lipogenic genes (*Dgat2, Scd1, Srebp1c)*, and upregulated lipid catabolism genes (*Hsl*/*Acox*) expression. A similar pattern was observed in the DIO + EX group. The combination of DHA + exercise potentiated an increase in *Cpt1a* and *Ppara* genes, and AMPK activation, key regulators of fatty acid oxidation. Exercise, alone or in combination with DHA, significantly reversed the induction of proinflammatory genes (*Mcp1, Il6, Tnfα*, *Tlr4*) in DIO mice. DHA supplementation was effective in preventing the alterations induced by the HFD in endoplasmic reticulum stress-related genes (*Ern1/Xbp1*) and autophagy markers (LC3II/I ratio, p62, *Atg7*). In summary, long-term DHA supplementation and/or exercise could be helpful to delay NAFLD progression during aging in obesity.

## 1. Introduction

Obesity is a highly prevalent worldwide disease, which is associated with a number of metabolic diseases, such as non-alcoholic fatty liver disease (NAFLD) [1]. NAFLD encompasses a broad spectrum of physio-pathological conditions from simple steatosis, non-alcoholic steatohepatitis (NASH), liver fibrosis to cirrhosis [2]. Steatosis, the first stage of NAFLD, is characterized by ectopic triglyceride accumulation in hepatocytes. NAFLD development and evolution to NASH are commonly accompanied by a metabolic dysregulation and an increased systemic inflammation [3]. NAFLD has been considered as a global epidemic affecting 20–30% in the general population [4]. Aging is a critical risk factor for the development and progression of NAFLD [5]. With aging, fat redistribution occurs favoring the increase in abdominal (visceral) fat and the decrease in subcutaneous fat expansion, and promoting the ectopic accumulation of fat in liver, even in non-obese, non-diabetic subjects [6,7]. Of course, the prevalence of obesity and insulin resistance, which are considered as principal risk factors for developing NAFLD increases with aging, aggravating the progression of the disease [8,9].

Concerning the mechanisms that favour fat accumulation during aging, it has been suggested a role for endoplasmic reticulum (ER) stress [10,11]. Indeed, the ER is the major site of lipid synthesis in hepatocytes. Lipids are accumulated in hepatocytes as triglycerides (TG) through the esterification of fatty acids and glycerol by acyltransferase enzymes including diacylglycerol acyltransferase (DGAT), localized in the ER. Moreover, liver TG can be also generated by de novo lipogenesis (*DNL*), a process regulated by transcription factors such as the sterol regulatory element-binding proteins (SREBP), localized on the ER as membrane proteins after being synthesized [10]. Furthermore, X-Box Binding Protein 1 (XBP1), a key regulator of ER stress response, is a crucial player in hepatic lipogenesis. In fact, XBP1 deletion results in decreased liver lipid production [12]. Activated endoplasmic reticulum to nucleus signaling 1 (ERN1, also called IRE1α) cleaves XBP1 mRNA to produce a spliced variant (XBP1s) mRNA, which encodes a transcriptionally active form XBP1. Moreover, lipid composition of the ER membrane is thought to be important for direct activation of IRE1α in the absence of classical ER stress induced by unfolded proteins [13,14]. Interestingly, IRE1α-XBP1 signaling has been linked to hepatic steatosis, and inflammation in NAFLD [15].

On the other hand, autophagy is a catabolic process that plays an essential role in the maintenance of cellular and tissue homeostasis, by the degradation of dysfunctional proteins and organelles. Autophagy has been shown to be a key regulator in the control of lipid metabolism [16], and the degradation of lipids via autophagy (lipophagy) has been revealed as a key pathway for the regulation of intracellular lipid content in hepatocytes [17]. Impaired autophagy/lipophagy during aging can lead to excessive tissue lipid accumulation such as hepatic steatosis and progression of NAFLD [17,18,19]. Autophagy can be modulated by ER stress, and it has been described that impaired autophagic flux is associated with increased ER stress during NAFLD development [20]. The chronic low-grade inflammation associated to both aging (*inflammaging*) and obesity also seems to contribute to the development/aggravation of NAFLD progression [21]. Inflammation has also been related to ER stress triggering, and IRE1α has been proposed as a factor linking both processes [22]. Indeed, a recent study has shown that IRE1α stimulates, via XBP1, hepatocyte-derived extracellular vesicles that promote inflammation in mice with steatohepatitis [15]. Therefore, strategies to block this pathway might be useful to reduce liver inflammation in NASH.

Unhealthy diets rich in saturated fat, trans-fats, simple sugars, and animal proteins, as well as sedentary lifestyle have been directly related with the susceptibility of developing NAFLD [23,24,25]. Currently, there are no effective pharmaceutical therapies for the treatment of NAFLD, and dietary control and increased physical activity/exercise that lead to decreased body fatness are the main approaches aiming to tackle NAFLD [24,26].

With this regard, some preclinical and clinical studies have suggested that omega-3 polyunsaturated fatty acids (n-3 PUFA) from marine origin (eicosapentaenoic acid, EPA and docosahexaenoic acid, DHA) may be effective in the early stages of NAFLD (excluding steatohepatitis and more advanced NASH stages) [27,28,29,30]. It has been suggested that DHA is a major bioactive n-3 PUFA accumulating in tissues and is likely responsible for many of the in vivo beneficial effects of n-3 PUFA [31]. However, few studies have analyzed the differential effects of DHA and EPA on improving NAFLD development/progression. Suzuki-Kemuriyama et al. [32] found that EPA had a greater hepatic TG-lowering effect, while DHA had a greater suppressive effect than EPA on hepatic inflammation and reactive oxygen species generation, without differences in fibrosis in male mice with NAFLD. Depner et al. [33] reported that DHA has a greater ability to prevent hepatic inflammation, fibrosis, and oxidative stress compared to dietary EPA in a low-density lipoprotein receptor (LDLR) knockout mouse model of western diet-induced NASH; however, DHA supplementation does not promote full remission of diet-induced NASH [31]. Several clinical trials have also suggested that supplementation with DHA alone or in combination with EPA seems to be effective at lowering liver fat in NAFLD patients, but had little effect in attenuating liver fibrosis [2,30]. Overall, among n-3 PUFA, it seems that DHA might exert more beneficial effects targeting hepatic inflammation and oxidative stress in NAFLD. Several mechanisms have been proposed to be involved in the regulation of hepatic lipid metabolism by n-3 PUFA, including the modulation of transcription factors and enzymes implicated in the regulation of fatty acid oxidation (peroxisome proliferator-activated receptor alpha, PPARα, adenosine monophosphate-activated protein kinase, AMPK), and lipogenesis (SREBP-1c, diacylglycerol Acyltransferase2, DGAT2) [27,34,35]. Most of the studies analyzing the effects of n-3 PUFA supplementation on NAFLD have been performed during short-term periods (4–24 weeks) in young mice (starting at 5–14 weeks-old). However, to our knowledge, there are no studies characterizing the potential beneficial actions of long-term dietary supplementation (12 months) with n-3 PUFA on the amelioration of NAFLD in obese aged mice.

On the other hand, several studies have shown that hepatic fat content can be reduced by exercise even without weight loss [26,36]. Mechanistic studies have suggested that exercise could prevent fatty liver in high-fat-diet (HFD) fed rodents by targeting insulin resistance, mitochondrial function, fatty acid oxidation, AMPK activation, and autophagy regulation [37,38,39,40]. However, there is a lack of studies addressing the effects of long-term, moderate exercise initiated during midlife [41] on NAFLD under obesogenic conditions. Moreover, few studies have analyzed the combined actions of a diet rich in n-3 PUFA and physical exercise on NAFLD development/progression in the context of aging and obesity.

Therefore, the aim of this study was to investigate the single and combined effects of long-term feeding with a DHA-rich HFD and regular treadmill exercise on liver steatosis development during aging. Moreover, we aim to characterize the potential underlying mechanisms, including the effects on gene/proteins involved in hepatic lipid accumulation, ER stress, autophagy, and proinflammatory status.

## 2. Materials and Methods

### 2.1. Animal Models and Experimental Design

Seven-week-old female C57BL/6J mice were purchased from Harlan Laboratories (Barcelona, Spain). Animals were housed at the animal facilities of the University of Navarra under strict controlled conditions (22 ± 2 °C, with a 12 h light–dark cycle, relative humidity, 55 ± 10%). All experiments were performed according to national animal care guidelines, and with the approval of the Ethics Committee for Animal Experimentation of the University of Navarra (Protocol 113–15), in accordance with the EU Directive 2010/63/EU. After acclimation, mice were divided into two experimental groups: (1) Control (C) group (*n* = 10) fed a normal control diet containing as energy: 20% proteins, 67% carbohydrates, and 13% lipids (2014 diet, Harlan Teklad Global Diets, Harlan Laboratories, Indianapolis, IN, USA), and (2) Diet-induced obese (DIO) group fed a high fat saturated diet (HFD) containing as energy: 20% proteins, 35% carbohydrates, and 45% lipids (D12451 diet, Research Diets, Inc., New Brunswick, NJ USA). Animals were fed ad libitum with these two diets for 4 months. Afterwards, the DIO group was divided into 4 experimental groups: (1) DIO group (*n* = 10) that continued with the HFD up to 18 months; (2) DIO + DHA group (*n* = 6) fed up to 18 months with the HFD containing a DHA-rich n-3 PUFA concentrate from fish oil, replacing 15% wt/wt of dietary lipids (Research Diets Inc., New Brunswick, NJ, USA); (3) DIO + Exercise (DIO + EX) group (*n* = 8) fed with the same HFD than the DIO group in combination with a regular aerobic physical exercise training up to 18 months; and (4) DIO + DHA + EX group (*n* = 9) fed with the HFD containing the DHA-rich n-3 PUFA concentrate, in combination with a regular aerobic physical exercise training up to 18 months. The control group was grown to 18 months as well. The DHA-rich n-3 PUFA concentrate (SOLUTEX0063TG, containing 683.4 mg DHA/g, 46.7 mg EPA/g, with a total content of n-3 PUFA of 838.9 mg/g as TG) was provided by Solutex (Spain). Importantly, given that the DHA-rich n-3 PUFA concentrate contained mixed tocopherols (2 mg/g of Covi-ox^®^ T-79EU), the HFD of the DIO and DIO + EX groups (from month 6–18) was supplemented with the same amount of tocopherols mix [42]. The different HFDs (prepared by Research Diets, Inc., New Brunswick, NJ, USA) were vacuum-sealed in 2.5 kg plastic bags, and kept frozen (−20 °C) until used to avoid rancidity (information about the diets can be found in Table S1). At the end of the experimental period, mice were sacrificed after overnight fasting and liver and blood samples were collected. Livers were weighed and serum samples were kept at −80 °C for further analysis.

### 2.2. Training Protocol

The DIO + EX and DIO + DHA + EX groups were subjected to a treadmill exercise program (LE8710M, Panlab, Barcelona, Spain) from 6 until 18 months-old. Before the beginning of the exercise training, the mice were allowed to adapt to the treadmill by running for 10 min on 2 consecutive days (first day at 3 m/min; second day at 4.8 m/min). From months 6 to 10, the mice were subjected to a low intensity training program (3 m/min for 5 min, increased to 4.8 m/min for 5 min, and then reached a maximum of 7.2 m/min for 20 min at 0% slope). At 10 months of age (midlife), the number of sessions and the speed of the training were increased to 5 days per week during 5 weeks with the following protocol: running time and speed were started at 5 m/min for 5 min, increased to 8 m/min for 5 min, and then reached a maximum of 12 m/min for 20 min at 0% slope. During the next 7 months, the exercise protocol was maintained, but the number of sessions was reduced from 5 to 3 days a week [43]. The mice of the non-exercise groups were left on the treadmill, without running, for the same period as the exercise groups.

### 2.3. Determination of Liver TG Content

Liver pieces (100–200 mg) were homogenized in phosphate buffer 0.1 M pH = 7–7.4 to determine lipid content. The Folch method [44] was used to extract lipids and TG content was determined by Infinity Triglycerides Liquid Stable Reagent (Thermo Electron Corporation, Colorado Springs, CO, USA) following the manufacturer’s instructions. TG content was normalized to mg of protein. Protein concentrations were determined by the BCA method according to the manufacturer’s instructions (Pierce-Thermo Scientific, Rockford, IL, USA).

### 2.4. Biochemical Analysis

Serum alanine amino transferase (ALT) and aspartate amino transferase (AST) were measured after a 12-h fasting period using a Pentra C200 autoanalyzer following the manufacturer’s instructions (Roche Diagnostic, Basel, Switzerland).

### 2.5. Liver Histology

Liver pieces were fixed in 3.7–4.0% neutral formalin (pH 7.4) for 24 h, dehydrated with 70% ethanol, and embedded in paraffin. Five μm thick sections were deparaffinized and stained with hematoxylin-eosin (H&E). Liver images (magnification 40X) were taken with an Olympus microscope (CKX31SF, Olympus Corp., Tokyo, Japan) coupled to an Olympus C-5060 camera (Olympus Corp., Tokyo, Japan).

### 2.6. Analysis of mRNA Expression by Real-Time PCR

Total RNA from liver was extracted with TRIzol™reagent (Invitrogen, ThermoFisher Scientific, Waltham, MA, USA). RNA quality and concentrations were measured by Nanodrop Spectrophotometer ND1000 (Nanodrop Technologies, Inc. Wilmington, NC, USA). RNA (5 μg) was then incubated with DNase I (Life Technologies, Carlsbad, CA, USA) for 30 min at 37 °C and reverse transcribed to cDNA using the High-Capacity cDNA Reverse Transcription Kit (Applied Biosystems; Thermo Fisher Scientific) according to the manufacturer’s instructions. Real-time PCR was performed using the Touch Real-Time PCR System (C1000 + CFX384, BIO-RAD, Hercules, CA, USA). Expression of *fatty acid synthase* (*Fas*), *Srebp1c*, *stearoyl-coenzyme A desaturase 1* (*Scd1*), *Dgat2*, *hormone sensitive lipase* (*Hsl*), *acyl-coenzyme A oxidase* (*Acox*), *carnitine palmitoyltransferase 1a* (*Cpt1a*), *Ppara*, *autophagy-related gene 5* (*Atg5*), *autophagy-related gene 7* (*Atg7*), *Xbp1*, *Ern1*, *monocyte chemoattractant protein-1* (*Mcp-1*), *tumor necrosis factor alpha* (*Tnfα*), *interleukin-6* (*Il-6*), and *Toll-like receptor 4* (*Tlr4*) genes were determined using predesigned Taqman^®^ Assays-on-Demand (Applied Biosystems, CA, USA) or by Power SYBR Green PCR Master Mix (BIO-RAD) [45,46]. Primers were designed with Primer-Blast software (National Center for Biotechnology Information, Bethesda, MD, USA; https://www.ncbi.nlm.nih.gov/tools/primer-blast). Primers sequences or references are shown in Tables S2 and S3, respectively. *36B4* was used as the housekeeping gene. Relative expression of the specific genes was determined using the 2^−ΔΔCt^ method [47].

### 2.7. Western Blot Analysis

Liver extracts were homogenized with Ultra-Turrax T25 (IKA) for 15 s in 350 µL lysis buffer (8 mmol/L NaH_2_PO_4_, 42 mmol/L Na_2_HPO4, 1% sodium dodecyl sulfate (SDS), 0.1 mol/L NaCl, 0.1% NP40, 1 mmol/L NaF, 10 mmol/L sodium orthovanadate, 2 mmol/L phenylmethylsulphonylfluoride (PMSF), 10 mM ethylenediaminetetraacetic acid (EDTA), and 1% protease inhibitor cocktail 1 (MilliporeSigma, Darmstadt, Germany)). Then, the samples were centrifuged at 13,000 rpm for 15 min to obtain the supernatant fraction containing the proteins. The protein extracts were determined by the standardized method of Bradford (Bio-Rad Protein Assay; BIO-RAD). Proteins extracts (30–40 μg) were electrophoretically separated on 10–15% sodium dodecyl sulfate–polyacrylamide gel electrophoresis [48]. Then, proteins were electroblotted from the gel to polyvinylidene difluoride membranes (Amersham^TM^ Hybond^TM^, GE Healthcare Life Science, Freiburg, Germany) or nitrocellulose membranes (Amersham^TM^ Protran^TM^, GE Healthcare Life Science). Efficient protein transfer was monitored by Ponceau S stain. Next, membranes were blocked (5% bovine serum albumin [BSA]) for 1 h at room temperature and probed with specific primary antibodies, at 1:1000 overnight at 4 °C in 1% BSA, against AMPK (rabbit, 2532), phospho-AMPK (rabbit, 2535), microtubule-associated protein 1A/1B-light chain 3 (LC3, rabbit, 4108) (Cell Signaling Technology, Danvers, MA, USA), p62 (rabbit, P0067) and β-actin (mouse, A1978) (Sigma-Aldrich). Thereafter, infrared fluorescent secondary antibodies anti-rabbit (Cell Signaling Technology, 5366S) and anti-mouse (LI-COR Biosciences, Lincoln, NE, USA, 926–32210) were used for p-AMPK/AMPK and quantitated using an Odyssey Sa infrared imaging system (LI-COR). For p62, LC3, the immunoreactive proteins were detected with enhanced chemiluminescence (Thermo Fisher Scientific, Waltham, Mass., USA) using with the corresponding peroxidase conjugated secondary antibody at 1:10,000 and quantified by densitometry analysis (Imagen Studio Lite; LI-COR Biosciences, Lincoln, NE, USA). The results are expressed in relation to the control value, which was set to 100%.

### 2.8. Statistical Analysis

Statistical analyses were performed using GraphPad Prism 9 software (Graph-Pad Software, La Jolla, CA, USA). Data are presented as mean ± SEM. Differences between groups were set up as statistically significant when *p* value was lower than 0.05. Comparisons between the values for different variables were analyzed by one-way ANOVA followed by Tukey post hoc test once the normality had been screened using Kolmogorov–Smirnov and Shapiro–Wilk tests. Researchers were not blinded to the testing condition during experimental testing.

## 3. Results

### 3.1. Effects of Long-Term DHA Supplementation and Exercise on NAFLD Features in 18-Month-Old DIO Mice

**Figure 1:**
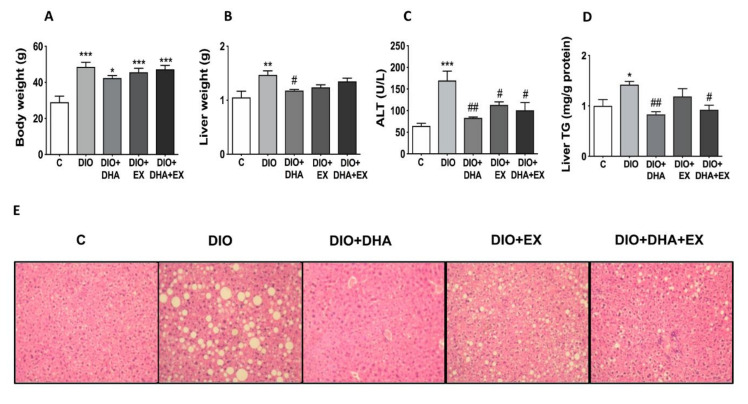
Effects of long-term DHA supplementation and/or aerobic exercise training on: (**A**) body weight, (**B**) liver weight, (**C**) serum ALT, (**D**) liver triglyceride (TG) content, and (**E**) liver morphology in 18-month-old diet-induced obese (DIO) mice. Data are mean ± SEM. *n* = 6–10 animals/group. * *p* < 0.05, ** *p* < 0.01, *** *p* < 0.001 vs. C group. ^#^ *p* < 0.05, ^##^ *p* < 0.01 vs. DIO group.

As shown in Figure 1A, the long-term feeding with the HFD (DIO group) induced a significant increase in body weight, which was not significantly reduced by any of the treatments (DHA, exercise or the combination of both). However, all the treatments partly reduced the increase of liver weight observed in the DIO group, being significant in the DHA supplemented group (Figure 1B).

Fatty liver development and liver function was also assessed by quantification of the serum transaminases ALT and AST, histological analysis of liver, and determination of hepatic TG content. Serum ALT levels were significantly increased in the DIO group, and interestingly all interventions (DHA, exercise or the combination of both) were able to significantly reduce the circulating ALT levels (Figure 1C), although no changes were found on AST (data not shown). Interestingly, the DHA supplementation, alone or in combination with exercise, significantly lowered the increased liver TG content observed in DIO mice (Figure 1D). Exercise training alone had a more moderate effect on the reduction of liver lipid content. Thus, the significant increase in liver TG content observed in DIO mice was not observed in DIO + EX mice as compared to control mice although significant differences between DIO and DIO + EX group were not reached (Figure 1D). Consistent with the biochemical analysis, the liver histology analysis showed that the 18-month-old DIO mice developed a more pronounced liver steatosis than age-matched control-fed mice as shown by the presence of macro- and microvesicular steatosis with nuclear displacement. Interestingly, the supplementation with DHA, alone or in combination with exercise, induced an amelioration of liver steatosis as shown by the almost absence of macrovesiclular steatosis and presenting some microvesicular lipid droplets without disturbance of nucleus (Figure 1E). All these data suggest that long-term DHA supplementation and exercise training can be useful to attenuate the development of NAFLD in DIO mice during aging.

### 3.2. Effects of Long-Term DHA Supplementation and Exercise on Hepatic Lipid Metabolism Regulators in 18-Month-Old DIO Mice

In order to characterize the mechanisms underlying the anti-steatotic effects of DHA supplementation alone or in combination with exercise, we next analyzed the expression levels of master genes involved in the control of lipid accumulation (fatty acid esterification and *DNL*) as well as lipid catabolism (lipolysis and fatty acid oxidation) in the liver.

**Figure 2:**
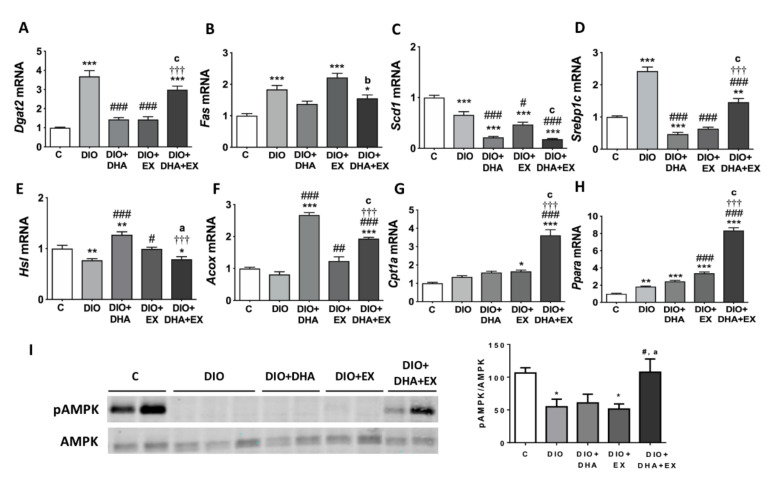
Long-term DHA supplementation and/or aerobic exercise training regulates the expression of lipid metabolism related genes/proteins in liver of 18-month-old DIO mice. (**A**–**H**) mRNA expression levels of genes involved in hepatic lipogenesis (**A**–**D**), lipolysis (**E**) and fatty acid oxidation (**F**–**H**). (**I**) Representative Western blot and densitometry analysis of AMPK phosphorylated and total (left panel). Band densities of phosphorylated AMPK were normalized by total AMPK (right panel). Data are expressed as mean ± SEM. *n* = 6–10. * *p* < 0.05, ** *p* < 0.01, *** *p* < 0.001 vs. C group. ^#^ *p* < 0.05, ^###^ *p* < 0.001 vs. DIO group. ^†††^ *p* < 0.001 for DIO + DHA vs. DIO + DHA + EX group. ^a^ *p* < 0.05, ^b^ *p* < 0.01, ^c^ *p* < 0.001 for DIO + EX vs. DIO + DHA + EX group.

DGAT2 catalyzes the final step in triglyceride synthesis and play a key role in hepatic TG content [49]. As expected, the HFD elevated the expression level of *Dgat2* gene, which was reversed by DHA supplementation or regular aerobic exercise; however, the ameliorative effect did not reach significance in the combined therapy group (Figure 2A). *DNL* is a complex and highly regulated pathway, which involves the participation of several key enzymes such as FAS and SCD1 [50]. DIO mice exhibited increased expression of *Fas* mRNA, which was partly prevented in the DHA-supplemented groups (Figure 2B). In addition, the expression of *Scd1*, which converts saturated fatty acids to monounsaturated fatty acids [51], decreased significantly in the three intervention groups compared to the DIO group. Noteworthy, the decrease in the groups supplemented with DHA was much more predominant than in the exercise training alone (Figure 2C). SREBP1c is the main transcription factor regulating *DNL* [52]. In our study, a dramatic upregulation of *Srebp1c* mRNA was observed in the DIO group compared with the control group, while treatment with DHA, exercise, or both decreased it, although the combined therapy had a lower effect when comparing with the DHA or exercise alone-treated groups (Figure 2D).

The catabolism of hepatic lipids was estimated by the expression of genes involved in lipolysis (*Hsl*) [53] and fatty acid oxidation (*Cpt1a* and *Acox*) [54]. The decreased mRNA expression level of *Hsl* induced by the HFD was remarkably upregulated by DHA supplementation or regular aerobic physical exercise alone, but not by their combination (Figure 2E). In terms of fatty acids oxidation, the mRNA expression of *Aco*x tended to be lower in untreated DIO mice and interestingly, the supplementation with DHA alone or in combination with exercise, caused a prominent upregulation of this gene involved in the peroxisomal β-oxidation of fatty acids (Figure 2F). Figure 2G shows that *Cpt1*a mRNA levels, involved in the mitochondrial β-oxidation process, were significantly upregulated in DIO mice performing regular aerobic physical exercise group and especially in the group that combined exercise with DHA supplementation, suggesting a synergistic effect of both interventions. We also analyzed the expression of *Ppara*, which encodes for a transcription factor that targets genes involved in hepatic fatty acid oxidation [55]. It was interesting to note that *Ppara* mRNA expression was upregulated in DIO mice, which is in agreement with previous observations [56]. This upregulation of *Ppara* by the HFD could be related to the increased need to oxidize fatty acids since higher levels of fatty acids arrive at the liver; however, this upregulation of fatty acids oxidation seems to be insufficient to catabolize the extra lipid loads in DIO mice. Interestingly, our data show that *Ppara* gene expression was significantly increased in the exercise trained groups and further upregulated in the group combining exercise with DHA supplementation (Figure 2H), which could contribute to the activation of hepatic liver catabolism and the improvement in liver steatosis observed in these groups. AMPK is a crucial nutrient and energy sensor that plays a key role in energy homeostasis maintenance. AMPK activation switches on catabolic pathways that generate ATP (such as fatty acid oxidation), while switching off biosynthetic pathways that consume ATP (i.e., TG synthesis) [57]. Furthermore, some studies have suggested that hepatic PPARα activity can be regulated also by AMPK, although conflicting results were found [58,59]. Here we evaluate the effects of exercise and/or DHA on AMPK phosphorylation, which leads to its activation [60]. Figure 2I shows that the phosphorylation of AMPK was significantly reduced in DIO mice, which is in agreement with previous studies in HFD-fed rodents [60]. Interestingly, only the combination of DHA and exercise was able to reverse the inhibitory effect of the HFD on AMPK activation, which could also account for the reduced liver steatosis since hepatic AMPK activation has been shown to lead to reduced steatosis and inflammation in obese mice [61].

### 3.3. Effects of Long-Term DHA Supplementation and Exercise on Hepatic Pro-Inflammatory and ER-Stress Related Genes in 18-Month-Old DIO Mice 

**Figure 3:**
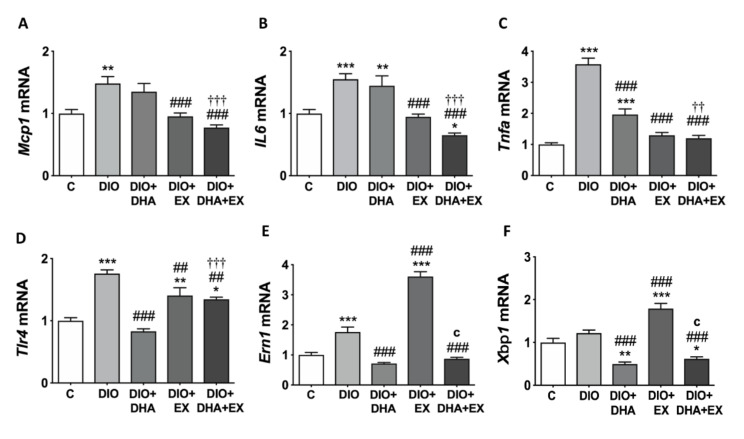
Long-term DHA supplementation and/or aerobic exercise training modulates the expression of pro-inflammatory genes (**A**–**D**) and of ER stress-related IRE1α-XBP1 pathway genes (**E**,**F**) in the liver of 18-month-old DIO mice. Gene expression values were normalized to control group. Data are mean ± SEM. *n* = 6–10. * *p* < 0.05, ** *p* < 0.01, *** *p* < 0.001 vs. C group. ^##^ *p* < 0.01, ^###^ *p* < 0.001 vs. DIO group. ^††^ *p* < 0.01, ^†††^ *p* < 0.001 for DIO + DHA vs. DIO + DHA + EX group. ^c^ *p* < 0.001 for DIO + EX vs. DIO + DHA + EX group.

*Mcp1*, *Il6*, *Tnfα* and *Tlr4* levels are key indicators of the proinflammatory state in liver [62]. All these genes were significantly upregulated in DIO mice compared with control group. The exercise intervention alone or in combination with DHA was able to reverse the induction of all these proinflammatory genes, while DHA only attenuated the expression of *Tnfα* and *Tlr4* (Figure 3A–D).

Mutual interaction between inflammation and ER stress seem to play important roles in NAFLD pathogenesis [10]. ER stress is regulated partially through IRE1α-XBP1 pathway [63,64], which has been related with promotion of inflammation in NASH [15]. Our current results show that the expression of *Ern1*, the gene that encodes for IRE1α, was upregulated in DIO mice compared with control mice (Figure 3E). DHA supplementation alone or combined with exercise significantly downregulated *Ern1* and *Xbp1* expression. However, regular treadmill exercise alone predominantly upregulated the expression of both *Ern1* and *Xbp1* in relation to other experimental groups (Figure 3E,F).

### 3.4. Effects of Long-Term DHA Supplementation and Exercise on Hepatic Autophagy Regulators in 18-Month-Old DIO Mice

**Figure 4:**
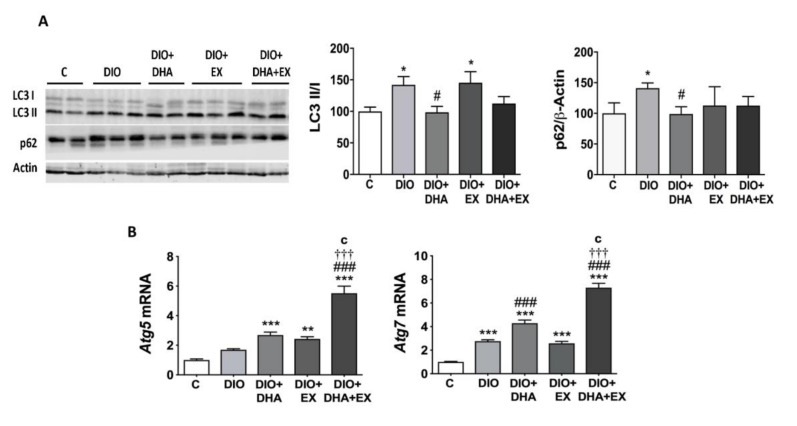
Long-term DHA supplementation and/or aerobic exercise training regulates the expression of key autophagy-related proteins/genes in the liver of 18-month-old DIO mice. (**A**) Representative Western blot (left panel) and densitometry analysis (right panels) of LC3 I and II and p62. Band densities of p62 were normalized to β-actin. (**B**) mRNA expression levels of hepatic *Atg5* and *Atg7*. Data are expressed as mean ± SEM. *n* = 6–10. * *p* < 0.05, ** *p* < 0.01, *** *p* < 0.001 vs. C group. ^#^ *p* < 0.05, ^###^ *p* < 0.001 vs. DIO group. ^†††^ *p* < 0.001 for DIO + DHA vs. DIO + DHA + EX group. ^c^ *p* < 0.001 for DIO + EX vs. DIO + DHA + EX group.

The impairment of autophagy has been also shown to play an important role in the hepatic lipid metabolic disorder contributing to NAFLD during obesity and aging [65]. Autophagy activity was assessed by the LC3-II/LC3-I ratio, the levels of p62, and the mRNA expression of *Atg5* and *Atg7* (Figure 4A,B). The HFD feeding induced a significant increase in the LC3-II/LC3-I ratio as compared to the control group, while DHA supplementation alone or combined with regular aerobic physical exercise was able to reverse this response returning the ratio to similar levels to that observed in the control group (Figure 4A). The upregulation in LC3-II/LC3-I indicates an increased number of autophagosomes, but this does not always mean upregulation of the autophagic flux, as it can occur as a consequence of a reduced degradation of autophagosomes [66]. The autophagic flux can be measured by the change in LC3-II levels in the presence vs. absence of lysosomal inhibitors, which measures the rate of LC3-II degradation [20]. However, these cannot be measured in mice liver samples, and therefore, we evaluated the levels of p62, a selective substrate of autophagy which is decreased when the autophagic flux is activated and vice versa, as previously described [20]. Our data showed an increase in p62 protein in DIO mice, which suggests an inhibition of the autophagic flux, even if the ratio LC3II/I is increased. Interestingly, this increase in p62 levels in DIO mice was prevented by DHA supplementation (Figure 4A), suggesting a normalization of the autophagic flux to almost control levels.

Finally, we also tested if the DHA supplementation and/or exercise could modify the expression of autophagy-related genes *Atg5* and *Atg7* [67]. Our data show that both groups supplemented with DHA exhibited higher levels of *Atg7*, especially when combined with exercise (Figure 4B). The combination of DHA and exercise also caused a significant upregulation of *Atg5* mRNA levels (Figure 4B). Our findings suggest that DHA supplementation, alone or combined with exercise, seems to be effective in promoting and/or restoring liver autophagy pathways altered by the HFD.

## 4. Discussion

NAFLD is a progressive degenerative process related to aging [68]. During aging, hepatic blood flow often decreases, lipid anabolism/catabolism is impaired, and inflammation and ER stress are induced [69,70]. These physio-pathological changes also occur in NAFLD progression [71,72,73]. In this study, the C57BL/6J female mice model was used because of its susceptibility to develop obesity and a NAFLD-like phenotype when fed a HFD [74,75]. In our study, we also aimed to characterize how an obesogenic diet can promote the NAFLD development and progression that naturally occurs during aging. As expected, feeding with a diet high in saturated fat and sucrose drastically worsened the signs of liver steatosis associated with aging, in agreement with previous studies [76,77].

While some trials in humans have suggested that total calorie consumption, rather than dietary fat composition, seems to be an important factor in NAFLD onset [78], several preclinical studies have shown that the type of fat could be a key determinant of the risk to develop NAFLD, suggesting a protective role for n-3 PUFA [31,34,79,80,81,82,83]. However, whether dietary n-3 PUFA levels influence NAFLD risk independently of total calorie intake and changes in body weight, especially during aging, remains unclear. In this way, our current data clearly show that chronic feeding with a HFD (45%) in which part of the saturated fat was replaced with a DHA-rich n-3 PUFA concentrate markedly attenuated NAFLD development, even without significantly reducing body weight in obese 18-month-old female mice.

Regarding the mechanisms by which the dietary DHA could ameliorate the aggravation of NAFLD induced by the diet high in saturated fat during aging, our data revealed that mice receiving dietary DHA showed a significant reduced expression of lipogenic genes such as *Dgat2*, *Scd1,* and *Srebp1c*. These data suggest that long-term dietary feeding (12 months) with DHA, even in the context of a HFD, modifies lipid liver metabolism balance, reducing the pathways favoring TG accumulation, and preventing the progression of NAFLD in obese mice during aging. These observations are in agreement with previous studies that have shown that shorter periods of n-3 PUFA (EPA and/or DHA) supplementation have beneficial effects on different experimental models of NAFLD in younger animals by decreasing the levels/activity of key regulators of hepatic fatty acid synthesis and lipogenesis, including FAS, SCD1, DGATs, and SREBP1c [27,35,79,80,84,85,86].

ER stress has been shown to play a key role in liver lipogenesis regulation and in the development of NAFLD during aging [10]. Several studies have suggested that the ER-induced IRE1α-XBP1 pathway seems to play an important role in the reprogramming of lipid metabolism. Upon ER stress, XBP1 is spliced by IRE1, generating functional XBP1 [87]. Furthermore, XBP1 is a crucial player in hepatic lipogenesis, since XBP1 deletion results in decreased liver lipid production [88]. Interestingly, our data show that DHA-fed mice exhibited a marked reduction in the mRNA expression of both *Xbp1* and *Ern1*, the gene that encodes for IRE1α, which could also account for the anti-lipogenic properties of this fatty acid in liver and thus the prevention of NAFLD in old obese mice. These data are in agreement with those observed by Kandeil et al. [89] evidencing a marked reduction of *Xbp1* mRNA levels and other ER stress biomarkers (CHREBP, CHOP, GRP78) in liver of young rats supplemented with n-3 PUFA, in parallel with the reduction of hepatic lipid content. The study of Gonçalves et al. [90] has also found reduced expression of hepatic *Xbp1* after dietary supplementation with the n-3 PUFA α-linolenic acid (ALA). Moreover, it has been shown that DHA ameliorates fructose-induced hepatic steatosis by modulating ER stress response (reducing *Xbp1* mRNA and pIRE1α and IRE1α levels) in primary mouse hepatocytes [91].

Our data also demonstrate that chronic DHA consumption upregulates the expression of genes involved in lipid catabolism (*Hsl* and *Acox*). Similarly, the trial of Fukunaga et al. [92] has observed that n-3 PUFA administration (in form of TG and phospholipids) to young Wistar rats has a significant stimulatory effect on *Acox* mRNA expression, the enzyme catalyzing the first step of peroxisomal β-oxidation. In contrast, only a moderate non-significant effect of chronic DHA intake was observed on the mRNA expression of *Cpt1a* (mitochondrial β-oxidation) and the transcription factor *Ppara* in the 18-month-old obese female mice. Another study has described that the increased hepatic fatty acid oxidation observed after n-3 PUFA supplementation is accompanied by the upregulation of both *Acox*, *Cpt1,* and *Cpt2* mRNA levels, but only ACOX and CPT2 activity were significantly increased [93]. However, other trials have shown that a relatively short term (11–12 weeks) of n-3 PUFA supplementation has the ability to significantly recover the fatty acid oxidation via *Ppara* activation, inducing *Cpt1* and *Acox* transcription [34,94].

Hepatic lipid catabolism can also take place through lipophagy [95]. Our data show that the HFD feeding induced a significant increase in hepatic LC3-II/LC3-I ratio, which occurred in parallel with increased p62 protein levels. These data are in agreement with previous observations in the liver of HFD-fed mice [20,96], and in the liver of patients with NAFLD, at both stages steatosis and NASH [20]. Although the upregulation in LC3-II/LC3-I might suggest an increased number of autophagosomes, the higher levels of p62, a selective substrate of autophagy, suggest that the autophagic flux is reduced in DIO animals [96]. Indeed, it has been described that the accumulation of LC3-II and autophagosomes, which was previously considered as a consequence of increased autophagy, could be in fact a consequence of a decreased autophagic flux [20,96]. Our current data show that dietary DHA supplementation reverses the increase induced by the HFD on LC3II/LC3I ratio and p62 protein expression, suggesting the ability of this n-3 PUFA to promote the autophagic flux that was blocked by the saturated fat. Few studies have analyzed the effects of n-3 PUFA on autophagy in hepatocytes. A previous study has reported that n-3 PUFA decreased lipid accumulation reversing lipotoxicity through the induction of autophagy in hepatocytes, which was related with the downregulation of *Scd1* expression [97].

ER stress can lead to the upregulation of many pro-inflammatory cytokines, including TNF-α, IL-1β, and IL-6. ER stress-induced inflammation can contribute substantially to disease progression from liver steatosis to NASH [10]. Our data revealed that DHA attenuated the overexpression of *Tnfα* and *Tlr4* found in the steatotic liver of DIO mice, suggesting that supplementation with this n-3 PUFA could be helpful to prevent/delay the progression of NAFLD during aging in obese mice. Several studies have demonstrated that shorter terms of supplementation with n-3 PUFA attenuated the exacerbation of pro-inflammatory cytokines induced by the HFD in younger mice [27,94,98].

Chronic aerobic exercise is a first-line treatment to mitigate NAFLD [99]. Thus, a recent review has suggested that in post-menopausal women and aged female mice, exhibiting increased risks of developing NAFLD, exercise training seems to exert a protective effect on both expression of genes involved in lipid accumulation and on expression of inflammatory genes in liver [100]. A number of studies conducted in young animals with NAFLD illustrated that the beneficial effects of short-medium exercise training periods on liver steatosis are conditioned to body weight loss [39,101,102,103,104,105,106]. However, other studies in young mice described a reduction in hepatic TG levels and lipid droplet size by short/medium-term exercise training (3 weeks or 16 weeks), without significant body weight loss [38,107,108]. Yet, the full mechanisms of the exercise-induced anti-steatosis effects remain unclear. Our study showed that long-term treadmill exercise training initiated in an adult age reduced serum ALT levels and prevented the significant increase in liver weight and liver TG content observed in 18-month-old DIO mice, even without significant changes in body weight loss. The reduced serum ALT level was also uncovered previously by Marques et al. [109], by training young mice with 8 weeks, moderate-intensity treadmill running.

Our study has also unraveled that long-term treadmill exercise significantly reduced the expression of lipogenic genes (*Srebp1c*, *Scd1,* and *Dgat2*), and upregulated genes related with lipid catabolism, especially fatty acid oxidation (*Acox* and *Ppara)*. These observations are consistent with previous studies showing that exercise (during 8–16 weeks) can regulate hepatic lipid metabolism by downregulating lipogenesis-related gene expression [39,105,106,110] and upregulating fatty acid oxidation [39,105,110,111,112] in younger mice. However, one study described that treadmill training for three weeks did not induce changes in *DNL* markers in non-obese mice with liver steatosis induced by a sucrose-enriched choline-deficient diet. However, a mild stimulatory effect on PPARα-mediated induction of fatty acid oxidation was observed [38]. Our current data suggest that both reduced lipogenesis and increased fatty acid oxidation could contribute to the beneficial effects of long-term exercise on liver steatosis markers.

In contrast, treadmill exercise did not seem to effectively reverse the alterations of autophagy markers observed in 18-month-old DIO mice. Our data are similar to the results obtained in a recent study in mice fed a HFD for 12 weeks and then subjected to 8 weeks of treadmill exercise, showing no significant changes on autophagy/lipophagy markers [90]. However, a previous study conducted in younger mice (8-week-old) fed a Western diet for 4 weeks, followed by 4 weeks of voluntary wheel running, showed a stimulatory action of exercise on autophagy characterized by greater LC3II/I ratio and lower p62 protein [40]. In addition, a recent study in rats observed that exercise and dietary intervention (switching to standard chow diet) ameliorate HFD-induced hepatic steatosis and liver aging trough the promotion of lipophagy [113]. On the other hand, our current data found an exacerbation of the mRNA expression of *Xbp1* and *Ern1*, which could be apparently contradictory with the downregulation observed on lipogenic genes. However, the unfolded protein response (UPR) is a dynamic cellular mechanism for reducing ER stress [114,115]. In this context, the study of Kristensen et al. [116] provides evidence for pathway-specific exercise-induced activation of the UPR in mouse liver. Moreover, Chapados and Lavoie [117] also observed that treadmill exercise training resulted in an increase of *Xbp1* mRNA levels and BiP/GRP78 in HFD-fed female Sprague-Dawley rats treated with a microsomal triglyceride transfer protein inhibitor. Interestingly, the inhibitor also promoted fatty liver, suggesting that the exercise-induced upregulation of UPR markers may constitute a protective mechanism against ER stress in the liver. In the current study, we only measured the gene expression levels of *Ern1* and *Xbp1*, and it would be also of interest to assess the phosphorylation of IRE1α, as a more direct measure of IRE1 activation in this process [118]. Although it was not the goal of our study, it would be also interesting to characterize if the UPR activation is specific to IRE1α, which might indicate lipid-based activation or if it also involves other branches of the UPR such as ATF6, which would suggest that that is more likely due to unfolded protein accumulation in the ER [119]. Therefore, future studies assessing ATF6 and/or PERK pathways would be useful to gain a mechanistic understanding of UPR activation in the liver in the context of obesity and aging and in the response to dietary DHA supplementation and/or exercise.

In relation to NAFLD-related inflammation, previous studies in young mice have reported that short-term exercise training decreases the expression of proinflammatory genes (*Tlr4, Tnfα, Mcp1)* [106], macrophage infiltration, and ameliorates fatty liver [108]. Interestingly, our current data clearly demonstrate that long-term exercise training is also beneficial to prevent the upregulation of proinflammatory genes (*Mcp1*, *IL6*, *Tnfα,* and *Tlr4*) observed in the liver of 18-month-old obese mice, being normalized almost to the control level. This suggests that moderate chronic exercise is helpful to prevent hepatic inflammation and therefore the progression to NASH in old obese mice, even without inducing dramatic changes on liver TG content.

Very little evidence exists about the beneficial effects of combining n-3 PUFA supplementation and treadmill exercise training on NAFLD. A recent study in male obese Zucker rats (5–6 weeks old) fed with an ALA-supplemented diet with or without exercise training for 4 weeks, showed that ALA and exercise alone influence divergent metabolic processes, and that combination of both exerted the greatest benefit on hepatic lipid accumulation [120]. Interestingly, our current data suggest that the combination of long-term DHA supplementation with treadmill exercise has some common and some differential effects that each individual intervention. The combination of both therapies showed a more potent effect on the induction/activation of genes/proteins involved in fatty acid oxidation such as *Cpt1a*, *Ppara,* and AMPK and on lipophagy-related genes (*Atg5, Atg7*). These findings suggest a possible interactive and additive beneficial effect of long-term DHA supplementation and treadmill exercise on the lipid catabolism pathway and inflammation in NAFLD during aging, probably mediated by AMPK activation. AMPK is a key energy sensor protein [57] that has been shown to promote lipid catabolism by inhibiting *DNL* and stimulating fatty acid oxidation [121]. AMPK activation has been also related to attenuation of ER stress [122] and induction of autophagy [123]. Thus, increasing AMPK activities has been considered as a therapeutic approach to improve NAFLD [124]. Although several studies conducted in younger mice models of NAFLD have illustrated that short- or medium-term n-3 PUFA supplementation [27,82,94,125,126], and exercise interventions [37,39,106,111,127,128] can increase AMPK activation, to our knowledge our study is the first addressing the combined effects of long-term n-3 PUFA supplementation and exercise training on AMPK activation in obese aged mice.

NAFLD has been described as a sexual dimorphic disease [129]. Several previous studies in humans, confirmed by a recent meta-analysis, have suggested that women have a lower risk of NAFLD than men, but, once NAFLD is established, women have a higher risk of advanced fibrosis [130]. Furthermore, it was found that prevalence of NAFLD increases with age in women (independent of weight gain or influence of metabolic syndrome), but not in men [131]. On the other hand, a study in mice suggested that female mice are more susceptible to NAFLD [132]. Taken into account all these previous observations and the fact that our study was designed to characterize the effects of aging and long-term effects of DHA supplementation and exercise in already obese mice with steatosis, we focused on female mice. However, considering the sex differences in susceptibility to develop NAFLD/NASH and some contradictory results obtained in different models of diet-induced fatty liver between male and female mice [132,133,134,135], we cannot conclude that similar outcomes would be observed if the study had been carried out in obese-aged male mice.

## 5. Conclusions

In conclusion, our data show that replacing part of saturated fat by a n-3 PUFA concentrate rich in DHA even in the context of a HFD, ameliorates the development of NAFLD associated to obesity and aging. Furthermore, long-term exercise training under obesogenic conditions also modulates hepatic lipid metabolism and markedly reduces inflammatory markers, suggesting that it could be beneficial to prevent the progression of liver steatosis to NASH. Our study has also unraveled that the combination of both interventions (diet rich in n-3 PUFA and physical exercise) might have some additional beneficial effects on the upregulation of fatty acid oxidation probably through AMPK activation, and therefore in the prevention of NAFLD development associated to aging and obesity.

## Data Availability

Not applicable.

## Supplementary Materials

The following are available online at https://www.mdpi.com/2072-6643/13/2/501/s1, Table S1 (Diets composition); Tables S2 and S3: Primers used for RT-PCR.
