# Supplementary material for: Effects of Long-Term DHA Supplementation and Physical Exercise on Non-Alcoholic Fatty Liver Development in Obese Aged Female Mice
Source: Nutrients. 2021 Feb 3;13(2):501. doi: 10.3390/nu13020501 (PMC7913512; doi:10.3390/nu13020501)
Supplement: Supplementary file 1 [file nutrients-13-00501-s001.pdf]

Supplementary Materials

Effects of long-term DHA supplementation and physical exercise on non-alcoholic fatty liver development in obese aged female mice

Jinchunzi Yang [1], Neira Sáinz[1], Elisa Félix-Soriano[1], Eva Gil-Iturbe[1], Rosa Castilla-Madrigal[1], Marta Fernández-Galilea[1,2], J Alfredo Martínez[1,2,3] and María J. Moreno-Aliaga[1,2,3,*]

Table S1. Diets composition.

Diet	Standard HFD		HFD with DHA-rich n-3 PUFA Concentrate (containing mixed tocopherols*)		Standard HFD (with same amount of mixed tocopherols added)**	
**Product #**	**D12451**		**D16112301**		**D16112302**	
%	**g**	**kcal**	**g**	**kcal**	**g**	**kcal**
Protein	23.7	20	23.7	20	23.7	20
Carbohydrate	41.4	35	41.4	35	41.4	35
Fat	23.6	45	23.6	45	23.6	45
Total		100		100		100
kcal/g	4.73		4.73		4.73	
Ingredient						
Casein, 30 Mesh	200	800	200	800	200	800
L-Cystine	3	12	3	12	3	12
Corn Starch	72.8	291	72.8	291	72.8	291
Maltodextrin 10	100	400	100	400	100	400
Sucrose	172.8	691	172.8	691	172.8	691
Cellulose, BW200	50	0	50	0	50	0
Soybean Oil	25	225	25	225	25	225
Lard	177.5	1598	147.1	1324	177.5	1598
High DHA Oil (Solutex 0063TG)*	0	0	30.4*	274	0	0
Mineral Mix S10026	10	0	10	0	10	0
DiCalcium Phosphate	13	0	13	0	13	0
Calcium Carbonate	5.5	0	5.5	0	5.5	0
Potassium Citrate, 1 H2O	16.5	0	16.5	0	16.5	0
Vitamin Mix V10001	10	40	10	40	10	40
Choline Bitartrate	2	0	2	0	2	0
Mixed tocopherols	0	0	0	0	0.061	0
Total	858.15	4057	858.15	4057	858.21	4057

*The DHA-rich n-3 PUFA concentrate contains 2 mg/g of mixed tocopherols to prevent oxidation. **A standard high fat diet (HFD) including the same amount of tocopherols contained in the HFD formulated with the DHA-rich concentrate was prepared. Diets were formulated and prepared by Research Diets Inc.

Table S2. Mouse primers sequences for SYBR GREEN real-time PCR.

Gene	Species	Forward primer sequence (5′- - 3′)	Reverse primer sequence (5′- - 3′)
36b4	*Mus musculus*	CACTGGTCTAGGACCCGAGAAG	GGTGCCTCTGGAGATTTTCG
Fas	*Mus musculus*	GCTGCGGAAACTTCAGGAAAT	AGAGACGTGTCACTCCTGGACTT
Srebp1c	*Mus musculus*	GGCCCGGGAAGTCACTGT	GGAGCCATGGATTGCACATT
Dgat2	*Mus musculus*	CCGCAAAGGCTTTGTGAAG	GGAATAAGTGGGAACCAGATCA
Acox	*Mus musculus*	CTATGGGATCAGCCAGAAAG	AGTCAAAGGCATCCACCAA
Cpt1a	*Mus musculus*	CACCAACGGGCTCATCTTCTA	CAAAATGACCTAGCCTTCTATCGAA
PPARα	*Mus musculus*	TCAGGGTACCACTACGGAGT	CTTGGCATTCTTCCAAAGCG
Atg5	*Mus musculus*	CGTGTATGAAAGAAGCTGATGC	ACGAAATCCATTTTCTTCTGGA
Atg7	*Mus musculus*	ATGCCAGGACACCCTGTGAACTTC	ACATCATTGCAGAAGTAGCAGCCA
Xbp1	*Mus musculus*	ATCAGCTTTTACGGGAGAAAACTC	CCATTCCCAAGCGTGTTCTT
Ern1	*Mus musculus*	GGTCCAATCGTACGGCAGTT	TCTCTCACAGAGCCACCTTTGTAG
Tnfa	*Mus musculus*	CATCTTCTCAAAATTCGAGTGACAA	TGGGAGTAGACAAGGTACAACCC
Tlr4	*Mus musculus*	TCGAATCCTGAGCAAACAGC	GTGGACGGGTCGATGTCAC
Hsl	*Mus musculus*	CTGCTTCTCCCTCTCGTCTG	CAAAATGGTCCTCTGCCTCT

Table S3. Mouse primers references for Taqman real-time PCR.

Gene	Species	Reference
Mcp1	*Mus musculus*	Mm00441242_m1
Il6	*Mus musculus*	Mm00446190_m1
Scd1	*Mus musculus*	Mm00772290_m1
